# Recurrent multifocal adult rhabdomyoma in an elderly woman diagnosed with Birt-Hogg-Dubé syndrome: A case report

**DOI:** 10.3389/fsurg.2022.1017725

**Published:** 2022-09-30

**Authors:** Ulrik Ørsø Andersen, Marie Røsland Rosenørn, Preben Homøe

**Affiliations:** ^1^ Department of Otorhinolaryngology and Maxillofacial Surgery, Zealand University Hospital, Køge, Denmark; ^2^ Department of Pathology, Zealand University Hospital, Roskilde, Denmark

**Keywords:** multifocal, recurrent, rhabdomyoma (MeSH), Birt-Hogg-Dubé (BHD), oral cavity

## Abstract

Birt-Hogg-Dubé syndrome (BHD) is a rare inherited autosomal dominant condition caused by a mutation in the tumor suppressor gene FLCN. This mutation predisposes the carrier to multiple pulmonary cysts, recurrent pneumothorax, renal tumors and benign skin tumors. Since the first description of BHD, additional clinical signs have been added to the syndrome and a large variety of benign tumors, such as rhabdomyomas (RM), have been linked with the FLCN gene mutation. RMs are rare tumors derived from striated muscle. The adult extracardiac rhabdomyoma occurs mostly in elderly patients, with a male predominance. It is most often located in the head and neck area and it has a potential of recurrence. We report a case of recurrent multifocal ARM in the oral cavity, present in an elderly woman with BHD and treated surgically. This could add support to RMs being linked to BHD.

## Introduction

Birt-Hogg-Dubé syndrome (BHD) is a rare inherited autosomal dominant condition caused by a mutation in the tumor suppressor gene FLCN. This condition predisposes to multiple pulmonary cysts, recurrent spontaneous pneumothorax, renal tumors and benign skin tumors. Since the first description of BHD, additional clinical signs have been added to the syndrome and a large variety of benign tumors, such as rhabdomyomas (RM), have been linked with the FLCN gene mutation (1). RMs are rare tumors derived from striated muscle and are topographically classified as cardiac or extracardiac. The adult extracardiac rhabdomyoma occurs mostly in elderly patients, with a male predominance. It is most often located in the head and neck area and it has a potential of recurrence (2). We report a case of recurrent multifocal ARM in the oral cavity, present in an elderly woman with BHD and treated surgically. This could add support to RMs being linked to BHD.

### Case description and diagnostic assessment rhabdomyoma in BHD patient

**Figure 1:**
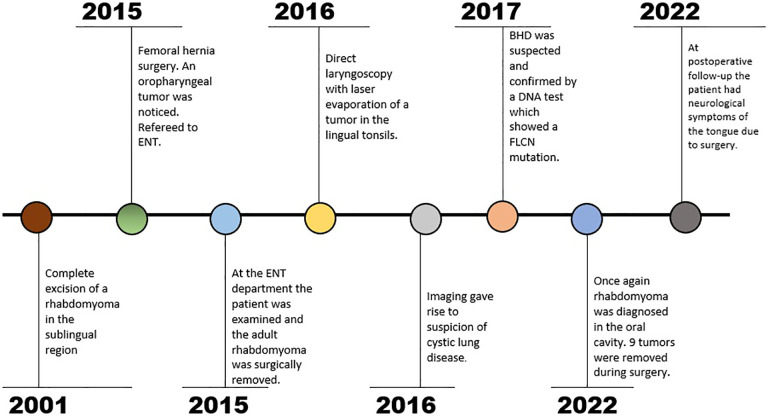
Text description: timeline.

An 87-year-old woman known with hypertension, wet macular degeneration, two episodes of pneumothorax (1978 and 1988) and previous excision of a rhabdomyoma in the sublingual region (2001, age 67 - the patient's medical record of this time is not available and further details or not available), had a femoral hernia surgery in 2015. During the intubation the anesthesiologist noticed an oropharyngeal tumor causing difficulties regarding the intubation. The patient was thereafter referred to the ENT department (See Figure 1 for timeline). She reported dysphagia but no other symptoms. At the examination a tumor of the basis of the left side of the tongue was seen covered with mucosa and without signs of malignancy. A magnetic resonance imaging (MRI) showed a homogenous tumor with contrast enhancement localized in the central tongue basis, with suspected growth into the surrounding tissue. The tumor was well-defined and was surgically removed *in toto* with free margins. Histologic examination showed a solid tumor composed of large tumor cells with eccentric located nuclei and cytoplasmic cross striations, consistent with RM.

At a clinical control a couple of months later another tumor was found at the left side of the base of tongue, again without ulcerations or any other signs of malignancy. The tumor was removed by direct laryngoscopy with laser evaporation. ARM was suspected but histology surprisingly showed tonsillar tissue without remaining components of RM. She was seen a month later with no symptoms and was completed from the ENT department.

Prior to the direct laryngoscopy a computed tomography (CT) thorax was performed due to suspicion of pneumothorax. The scan showed several pulmonal bullae and a high-resolution computed tomography (HRCT) gave suspicion of cystic lung disease. At this point her family three revealed a history of several cases of pneumothorax (grandmother, three aunts and two cousins). BHD was suspected and confirmed with a DNA test showing a pathologic mutation in FLCN gene (c.1285dup, NM_144997).

**Figure 2:**
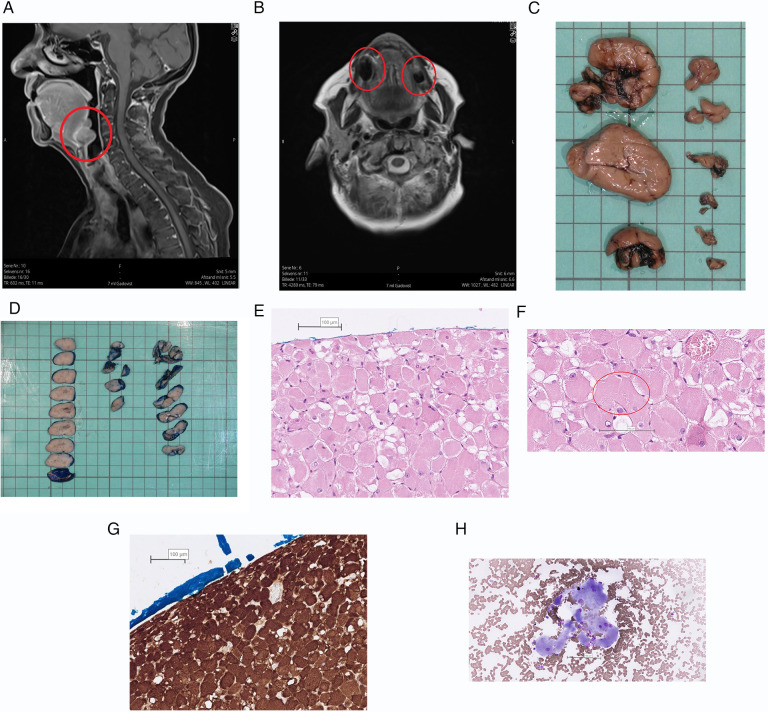
Text description: (**A**) MRI, T1, sagittal view. ARM marked with red circle. (**B**) MRI, T2, Axial view. ARM marked with red circle. (**C,D**) The specimen consisted of nine separate parts which had a diameter of 7 to 38 mm. The surface was well delineated and uniformly brown. The cut surface was solid and light brown with a few, small, darker areas. The surface of the largest tumors was stained blue before sectioning. (**E,F**) The growth pattern is solid and the tumor cells are large and polygonal with eccentric nuclei and abundant eosinophilic, granular and often vacuolated cytoplasm. The nuclei are rounded and uniform with light staining chromatin and a single nucleolus. Occasionally characteristic striations can be seen in the cytoplasm of the tumor cells. Stained with HE (Hematoxylin-Eosin). The scale bar is set at 100 µm. (**G**) The tumor cells show a strong cytoplasmic expression of Desmin by immunohistochemical staining, confirming the myogenic origin of the tumor. The scale bar is set at 100 µm. (**H**) The overall number of tumor cells in the specimen is sparse. The tumor cells are large (compared with adjacent erythrocytes) and have peripherally located nuclei with a single nucleolus and abundant, finely granular cytoplasm. Stained with MGG (Mey-Grünwald-Giemsa). The scale bar is set at 100 µm.

**Figure 3:**
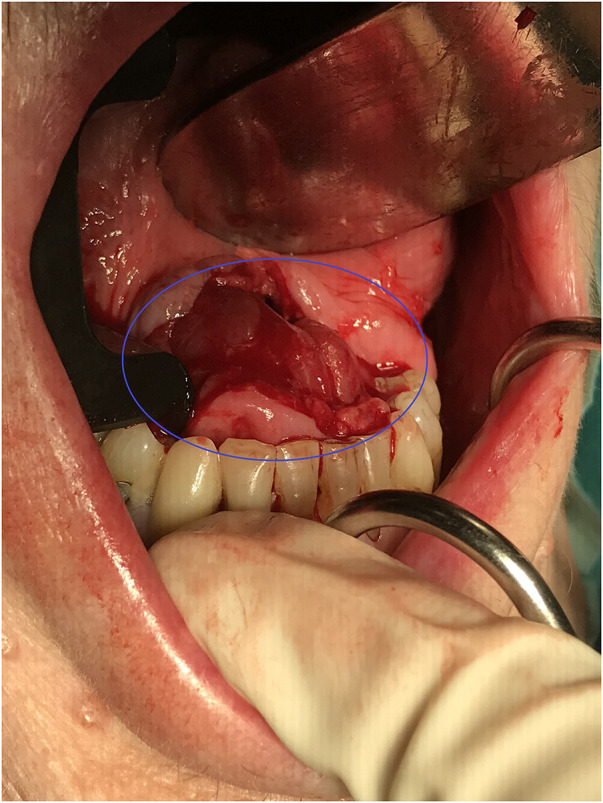
Text description: perioperative photos from the oral cavity showing an incision in the mucosa of the floor of mouth which gives direct contact with one of the RMs marked with a blue circle.

In early 2022 she was referred to the ENT department because of dysphagia and she had problems with speech due to a feeling of fullness in the bottom of her mouth. A large tumor was seen in the floor of mouth and another in the left side of the oropharynx. A fine needle aspiration from one of the tumors showed large, atypical cells resembling RM (See Figure 2H). MRI of the head and neck showed several tumors at the right side of the base of tongue and anteriorly at the base of tongue at the transition to the floor of mouth (see Figures 2A,B). Subsequently the tumors of the oral floor were completely excised but the tumor in oropharynx was not excised due to the lack of symptoms from this area (see Figure 3). During the surgery additional small tumors was discovered in the floor of mouth and as many as possible was surgically removed. At macroscopic examination the surface of the three largest tumors was alternately smooth and irregular (see Figures 2C,D). On histological examination the tumors showed solid growth of large tumor cells with abundant granular, eosinophilic and vacuolated cytoplasm and occasional cross striations (see Figures 2E–G). The histopathological diagnosis was RM with no sign of malignancy.

### Follow up

After the first surgery in 2015 the patient had oral pain which disappeared after the direct laryngoscopy in 2016. In 2022, the patient had a postoperative infection of the floor of mouth treated with intravenous and per oral antibiotics. At a later clinical control, the patient had a lack of sensation of the right side of the tongue, altered sense of taste and the food tended to accumulate anteriorly in the mouth. She was later diagnosed with fibrofolliculomas at a dermatologist and due to BHD. The patient is currently being monitored with scans of the kidneys at a medical department, where no signs of tumors have been found so far.

## Discussion

BHD is a very rare syndrome which is caused by an autosomal dominant mutation in the tumor suppressor gene FLCN. Patients with BHD are characterized by a wide phenotypic heterogeneity but most commonly presents with pulmonary cysts, recurrent pneumothorax, renal tumors and cutaneous tumors of the type fibrofolliculomas. Other clinical manifestations have been seen, such as different benign and malignant tumors, for example RM. It is still debated if they are a part of the syndrome (1). RMs are rare benign tumors which originate from striated muscle. They are classified as cardiac and extracardiac. The cardiac RM which mostly occurs in the heart of infants is usually linked to the tuberous sclerosis complex disease and has only once been reported one case of BHD (3). The extracardiac RM is subdivided into three types; adult, fetal and genital, of which the adult is the most frequent. ARM has a male predominance with and the median age is 65 years (2). They are mainly present in the head and neck region where the majority are located in the parapharyngeal space, larynx and the submandibular region. All of this fits with our case except from the gender (2). The most common clinical symptoms of ARM are globus sensation, hoarseness, a soft painless slow growing mass or dysphagia (2).

To determine the spatial localization of the tumor(s) CT and MRI are commonly used for imaging modalities. On MRI RM appears as isointense or hyperintense nodules with discrete enhancement upon gadolinium injection. On CT RM appears as an isodense tumor with discrete iodine contrast inhancement (2). The findings on MRI and CT are not specific for ARM and cannot be used in the diagnostics of RM but mostly to illuminate the anatomical relations. RM has a high uptake on F-FDG PET CT which could be used for anatomically localization of the RMs and to ensure complete removal of the multifocal RMs, but again the imaging modality is not specific for ARM (2). In our case the F-FDG PET CT could have predicted the large number of tumors and thereby potentially affected the planning of the surgery. For correct diagnosis a histologic examination is needed.

The standard treatment for ARM is surgical removal but with asymptomatic patients a watchfull waiting strategy is preferable when one has in mind that no previous malignant transformation of RM has been seen (2).

We conducted a Pubmed search on “rhabdomyoma and BHD syndrome (MESH terms)” and five cases occurred – see Table 1 (4–8). The localization of RMs in these case reports were the larynx, the paratracheal and paralaryngeal region, the heart, the sublingual and submandibular region – with a maximum of 3 tumors. Khalaf et al. illustrates that the localization varies a lot but is mainly within the head and neck area. In our search the mean age at presentation was 52 years – noted that one of the cases was a 5 years old child with a cardiac RM. The mean age of the cases with ARM was 61 years. The male to female ratio has been reported to be 13.5:1 but in our search the ratio was a 1:1 (2). This difference may be due to the small sample sizes.

**Table 1:** Table description: overview of the literature search.

Article	Year	Age at diagnosis	Sex	Localization	Laterality	Number of tumors (*n*)	Treatment	Presumptive diagnosis
Ulrik ØA, Preben H.	2022	67	Female	Base of tongue, submandibular space, floor of mouth	B	9	Resection submandibular gland, laser evaporation and surgical resection	–
Bajwa DS et. al. (4)	2021	63	Male	Left submandibular and bilateralt sublingual space	B	3	Unknown	–
Black M et al. (5)	2020	60	Male	Paratracheal and paralaryngeal next to the thyroid gland	U	1	Right neck mass resection	Thyroid nodule
Balakumar R et al. (6)	2018	51	Female	Larynx, right aryepiglottic fold	U	1	Microlaryngoscopy, excised with laser	–
Bondavalli D et al. (7)	2014	5	Male	Heart	U	2	Surgical resection	Tuberous sclerosis complex
Mikesell K et al. (8)	2014	67	Female	Paralaryngeal, next to the parathyroid gland	U	1	Surgical resection	Parathyroid adenoma

Our BHD case is the first with a recurrent history of RMs and the first with such a high number of tumors. A reason for the recurrent incidence could be that RMs are multilobulated and is overlooked perioperatively as seen in this case where several additional tumors were found during surgery. Khalaf et al. report recurrence of RM up to 10 years after the primary surgery in three out of fifteen cases.

The causal association between RMs and BHD in humans has yet to be established. Studies conducted on mice with inactive BHD genes suggest that the FLCN mutation causes an altered activity of the mTOR cellular pathway which regulates cell proliferation, autophagy and apoptosis. The mice phenotypically developed cysts and renal tumors similar to those found in BHD patients (9–11). In the BHD case of Bajwa et al. D.NA analysis were performed on the RM and they found loss of heterozygosity (LOH) at the site of the patients FLCNs pathogenic variant. Studies have shown the occurrence of LOH at the BHD locus in sporadic kidney tumors and tumors from patients with germline FLCN mutations (12, 13). All together this indicates that the FLCN mutation in the BHD could play a role in the tumorgenesis of RMs.

Our case and several other cases have diagnosed ARM in patients with BHD, indicating a potential link of the two. Our case is, to our knowledge, the first published case of multifocal and recurrent ARM in a patient with BHD and this should be a differential diagnosis when finding several RM tumors in the head and neck region.

## Data Availability

The original contributions presented in the study are included in the article/Supplementary Material, further inquiries can be directed to the corresponding author/s.
