# Corrigendum: Recurrent multifocal adult rhabdomyoma in an elderly woman diagnosed with Birt-Hogg-Dubé syndrome: A case report

**DOI:** 10.3389/fsurg.2022.1058498

**Published:** 2022-10-25

**Authors:** Ulrik Ørsø Andersen, Rosenørn Marie, Homøe Preben

**Affiliations:** Department of ear, Nose and Throat, Zealand University Hosptal, Køge, Denmark

**Keywords:** multifocal, recurrent, rhabdomyoma (mesh), Birt-Hogg-Dubé (BHD) syndrome, oral cavity

A corrigendum on Recurrent multifocal adult rhabdomyoma in an elderly woman diagnosed with Birt-Hogg-Dubé syndrome: A case report By Ulrik A, Marie R and Preben H. (2022) Front. Surg. 9: 1017725. doi: 10.3389/fsurg.2022.1017725

**Error in Figure/Table**

In the published article, there was an error in Figure 2 as published. One of the photos in the figure contains data that can possibly identify the patient. The corrected Figure 2 and its caption appear below.

**Figure 2:**
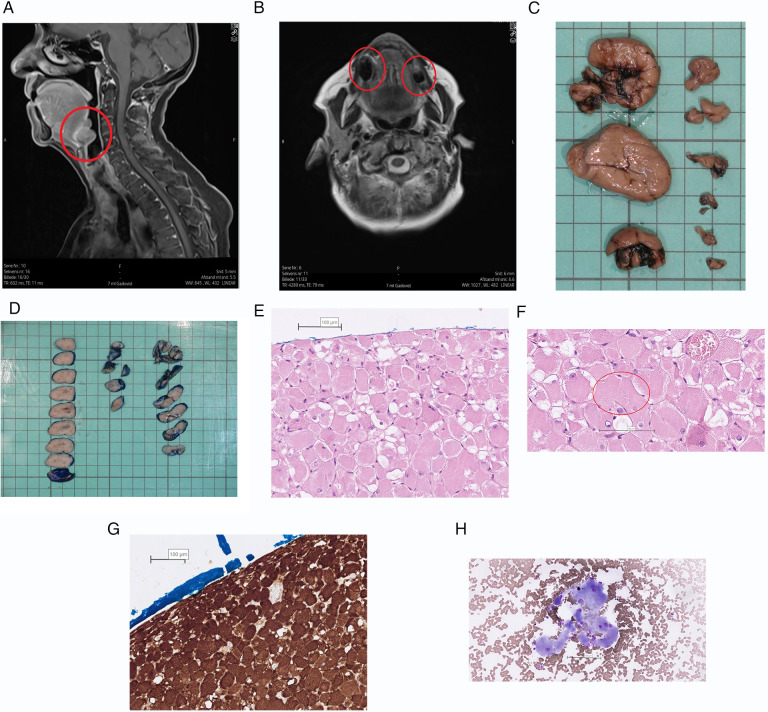
Text description: (**A**) MRI, T1, sagittal view. ARM marked with red circle. (**B**) MRI, T2, Axial view. ARM marked with red circle. (**C,D**) The specimen consisted of nine separate parts which had a diameter of 7 to 38 mm. The surface was well delineated and uniformly brown. The cut surface was solid and light brown with a few, small, darker areas. The surface of the largest tumors was stained blue before sectioning. (**E,F**) The growth pattern is solid and the tumor cells are large and polygonal with eccentric nuclei and abundant eosinophilic, granular and often vacuolated cytoplasm. The nuclei are rounded and uniform with light staining chromatin and a single nucleolus. Occasionally characteristic striations can be seen in the cytoplasm of the tumor cells. Stained with HE (Hematoxylin-Eosin). The scale bar is set at 100 µm. (**G**) The tumor cells show a strong cytoplasmic expression of Desmin by immunohistochemical staining, confirming the myogenic origin of the tumor. The scale bar is set at 100 µm. (**H**) The overall number of tumor cells in the specimen is sparse. The tumor cells are large (compared with adjacent erythrocytes) and have peripherally located nuclei with a single nucleolus and abundant, finely granular cytoplasm. Stained with MGG (Mey-Grünwald-Giemsa). The scale bar is set at 100 µm.

The authors apologize for this error and state that this does not change the scientific conclusions of the article in any way. The original article has been updated.

**Reminder:** Figures, tables, and images will be published under a Creative Commons CC-BY licence and permission must be obtained for use of copyrighted material from other sources (including re-published/adapted/modified/partial figures and images from the internet). It is the responsibility of the authors to acquire the licenses, to follow any citation instructions requested by third-party rights holders, and cover any supplementary charges.

